# Elevated levels of FN1 and CCL2 in bronchoalveolar lavage fluid from sarcoidosis patients

**DOI:** 10.1186/s12931-016-0381-0

**Published:** 2016-06-04

**Authors:** Carl Hamsten, Emil Wiklundh, Hans Grönlund, Jochen M. Schwenk, Mathias Uhlén, Anders Eklund, Peter Nilsson, Johan Grunewald, Anna Häggmark-Månberg

**Affiliations:** Immunology and Allergy Unit, Department of Medicine Solna, Karolinska Institutet and University Hospital, Stockholm, Sweden; Respiratory Medicine Unit, Department of Medicine Solna and CMM, Karolinska Institutet and Karolinska University Hospital, Stockholm, Sweden; Therapeutic Immune Design Unit, Department of Clinical neuroscience, Center for Molecular Medicine, Karolinska Institutet, Stockholm, Sweden; Affinity proteomics, SciLifeLab, School of Biotechnology, KTH – Royal Institute of Technology, Stockholm, Sweden

**Keywords:** Sarcoidosis, Bronchoalveolar lavage, Protein profiling, Löfgren’s syndrome

## Abstract

**Background:**

Sarcoidosis is a granulomatous systemic inflammatory disease in which more than 90 % of all patients develop pulmonary manifestations. Several gene associations have previously been described, but established and clinically useful biomarkers are still absent. This study aimed to find proteins in bronchoalveolar lavage (BAL) fluid that can be associated with the disease.

**Methods:**

We developed and performed profiling of 94 selected proteins in BAL fluid and serum samples obtained from newly diagnosed and non-treated patients with sarcoidosis. Using multiplexed immunoassays, a total of 317 BAL and 217 serum samples were analyzed, including asthmatic patients and healthy individuals as controls.

**Results:**

Our analyses revealed increased levels of eight proteins in sarcoidosis patients compared to controls. Out of these, fibronectin (FN1) and C-C motif chemokine 2 (CCL2) revealed the strongest associations. In addition, cadherin 5 (CDH5) was found to correlate positively with lymphocyte cell numbers in BAL fluid.

**Conclusions:**

Applying a high throughput proteomics screening technique, we found proteins of potential clinical relevance in the context of sarcoidosis.

**Electronic supplementary material:**

The online version of this article (doi:10.1186/s12931-016-0381-0) contains supplementary material, which is available to authorized users.

## Background

Sarcoidosis is a multi-organ inflammatory disorder characterised by tissue infiltration of mononuclear phagocytes and lymphocytes as well as noncaseating granuloma formation. Although sarcoidosis can affect any organ, more than 90 % of all patients exhibit pulmonary involvement [1]. Especially common in the northern part of Europe is the distinct subgroup of patients with Löfgren’s syndrome (LS), clinically characterised by an acute disease onset with bilateral ankle arthritis and/or erythema nodosum, fever and bilateral hilar lymphadenopathy with or without parenchymal infiltration [2]. While the cause behind sarcoidosis remains uncertain, antigen exposure in genetically susceptible individuals is believed to be a triggering factor [1]. A subgroup of patients develop chronic disease with fibrosis and eventually 5 % die of respiratory failure [3, 4]. Moreover, even without developing fibrosis, sarcoidosis can have a substantial impact on quality of life [5]. There is no specific treatment for sarcoidosis and no clinically established disease markers are available for diagnosis, monitoring of disease activity or prognosis, although genetic associations with the disease course have been described.

The diagnostic procedure in patients with suspected sarcoidosis in Sweden generally includes bronchoscopy for biopsies and BAL fluid retrieval, which presents an excellent opportunity for collecting and studying cells and soluble components such as proteins present at the affected site. Previous studies have investigated serum [6, 7], BAL fluid as well as BAL cells (macrophages) [8] in the search for potential markers of disease. However, comparative analysis of BAL fluid and serum from the same individuals has so far not been extensively analysed.

In this study, we performed protein profiling of BALF and serum using the antibody suspension bead array technology with the aim to find proteins associated to sarcoidosis. These bead arrays currently allow profiling of up to 384 analytes in 384 samples simultaneously, only using a few microliters of sample. Through the antibodies generated within the Human Protein Atlas, an initiative aiming to produce antibodies to all human proteins (www.proteinatlas.org), the arrays can be customized depending on the context and they have previously been applied in studies on various diseases and sample materials [9, 10].

## Methods

### Sample collection, processing and clinical information

In total, 251 sarcoidosis patients were included in the study and bronchoscopy with BAL was performed as a part of the initial diagnostic routine investigation as previously described [11]. BAL samples were obtained and immediately put on ice and brought to the laboratory where the samples were processed, aliquoted and stored at −80 until further processing. All samples were treated identically and there was no repeated freezing and thawing of the samples. Patients had an active disease, defined as patients with ongoing symptoms compatible with sarcoidosis like fever, fatigue, coughing and chest pain, and/or progression on chest radiography and/or deterioration of lung function and were not on treatment with immunosuppressive drugs. They were diagnosed according to the ATS/ERS/WASOG criteria [12] and further sub-grouped into those with Löfgren’s syndrome (“LS”; *n* = 131) or those without (“non-LS”; *n* = 120). As controls, both patients with mild allergic asthma (*n* = 17) as well as healthy subjects (*n* = 49) were included and bronchoscopy with BAL performed according to the same protocols as for the patients. Samples from patients with mild allergic asthma were obtained out of season. All patients only occasionally used bronchodilators but no corticosteroids or other immunosuppressive drugs. Healthy individuals were defined and selected as never-smokers, with no respiratory infections within 6 weeks prior to sample collection and with normal lung function tests and chest x-rays. Serum samples were obtained at the time of bronchoscopy and immediately frozen until analyzed. The study was approved by the local ethical committee and informed consent was obtained from all subjects. BAL fluid analyses during discovery and verification were conducted with concentrated samples prepared by thawing of crude BAL fluid (4 ml) in cold water followed by ultrafiltration using Amicon Ultra-4, PLBC Ultracel-3 Membrane, 3 kDa tubes (Millipore Amicon, Cork, Ireland) in a fixed angle rotator at +4 °C and 4000 × g to obtain samples with an end volume of 100–150 μl. The samples were then aliquoted into 96-well plates and stored at −80 °C until further use. Final protein concentration was assessed using bicinchoninic acid (BCA) spectrophotometer analysis (SoftMaxPro) and only samples with a total protein concentration below 10 mg/ml was included in the study. For comparison purposes, a set of 74 unprocessed BAL samples were also analysed.

A subset of 68 BAL samples from 35 sarcoidosis patients, 17 asthmatic patients and 16 healthy controls, was used to adapt the suspension bead array procedure for BAL fluid analysis. These, together with the remaining 249 BAL (216 sarcoidosis patients and 33 healthy controls) and 79 serum samples, were then profiled for discovery of disease-associated protein profiles. The levels of selected proteins were further investigated using all 317 BAL and an additional set of 138 serum samples. Clinical information on the included subjects is summarized in Tables 1 and 2.

**Table 1:** Sample demographics divided by subgroups and analysis stage

Group	N	Age	Gender	Chest radiographic	Smoking
		Median (Range)	M/F	Stage 0/I/II/III/IV	Non/Ex/Current
Discovery					
Asthma	17	22 (18–52)	6/11	NA	17/0/0
Control	16	24 (20–56)	8/8	NA	14/2/0
Löfgren’s syndrome	18	41 (30–57)	12/6	1^a^/8/9/0/0	11/5/2
Non-Löfgren’s syndrome	17	47 (29–67)	10/7	1^a^/5/8/1/2	6/8/3
Verification					
Control	33	25 (18–56)	18/17	NA	32/1/0
Löfgren’s syndrome	112	37 (23–62)	59/53	2^a^/72/38/0/0	60/33/18
Non-Löfgren’s syndrome	104	44 (26–77)	63/41	5/41/40/14/4	63/30/11

Complete information was not available for all patients

*NA* not applicable

^a^Patients had previously manifested lymphadenopathy, though determined to have fully regressed on conventional x-ray at the time of bronchoscopy

**Table 2:** Additional clinical information regarding lung function and BAL cell content

Group	VC		FEV1		DLCO		Macrophages		Lymphocytes		Neutrophils		Eosinophils		CD4/CD8 ratio	
	N	[%]	N	[%]	N	[%]	N	[%]	N	[%]	N	[%]	N	[%]	N	
Discovery																
Asthma	17	107 (65–123)	17	103 (82–126)	0	NA	17	86 (70–95)	17	11 (4–26)	17	1.4 (0–5.4)	17	0.8 (0–13)	0	NA
Control	1	110 (110–110)	16	114 (86–130)	0	NA	16	88 (66–97)	16	7 (2–28)	16	1.8 (0–6.8)	16	0.2 (0–1.4)	13	2 (1–5)
Löfgren’s syndrome	12	88 (81–96)	12	90 (66–100)	10	95 (62–139)	18	74 (48–94)	18	22 (6–46)	18	1 (0–5.8)	18	0.1 (0–1.6)	18	8 (1–26)
Non-Löfgren’s syndrome	13	81 (66–104)	14	79 (48–104)	10	80 (53–128)	17	75 (42–97)	17	23 (3–58)	17	0.8 (0–15.2)	17	0 (0–1.2)	17	6 (2–25)
Verification																
Control	9	108 (76–140)	14	106 (65–135)	0	NA	14	89 (60–96)	14	9 (3–39)	14	0.9 (0.4–2.2)	14	0 (0–0.6)	8	7 (1–37)
Löfgren’s syndrome	85	91 (57–118)	87	90 (52–120)	72	89 (56–126)	101	74 (34–98)	101	23 (2–65)	101	1 (0–14.4)	100	0.2 (0–13.3)	100	6 (1–33)
Non-Löfgren’s syndrome	80	86 (53–124)	87	87 (32–140)	59	87 (48–136)	96	75 (22–96)	96	23 (2–75)	96	1.4 (0–14.6)	96	0.2 (0–7.6)	94	5 (0–31)

The number of individuals indicate the availability of data for the respective parameters. Data represented as median (minimum-maximum) unless stated otherwise

*VC* vital capacity, *FEV1* forced expiratory volume in one second, *DLCO* diffusion capacity of the lung for carbon monoxide, *NA* not analysed

### Antibody selection and immobilization

Target proteins were selected through literature mining based on involvement in inflammatory processes, lung function or suggested relation to sarcoidosis. Antibodies for screening were obtained through the Human Protein Atlas and 96 antibodies towards 94 unique proteins were used for the initial discovery phase. Antibodies were immobilized onto color-coded, magnetic beads (MagPlex, Luminex corp.) as previously described [13]. In short, antibodies were diluted to 1.6 μg/ml in MES (2[N-Morpholino]-ethanesulponic acid) and covalently linked to the NHS/EDC activated carboxyl surface of beads, with a specific antibody immobilized to each bead identity. After removal of unbound antibodies and blocking of the bead surface, all identities were combined into an array in suspension. Four internal controls were included, with immobilised anti-human IgG (Jackson ImmunoResearch, 309-005-082) and anti-albumin (Dako, A0001) as positive controls while rabbit IgG from non-immunized rabbits (Bethyl) and a bead identity with no proteins immobilised served as negative controls.

### Assay procedure

The previously published plasma and CSF profiling protocols [13, 14] was adapted for analysis of BAL fluid protein profiling. In summary, both concentrated and crude BAL samples were diluted 1:2 in PBS supplemented with bovine serum albumin (BSA, Sigma, at an end concentration of 0.5 % (w/v) in PBS) and labelled with a ten fold molar excess biotin over total protein amount (approximated to 6 mg/ml) using a liquid handler (Selma, CyBio). Labelled samples were further diluted 1:8 in assay buffer (0.5 % (w/v) polyvinylalcohol, 0.8 % (w/v) polyvinylpyrrolidone and 0.1 % (w/v) casein (all SigmaAldrich) in 1xPBS supplemented with 10 % (v/v) rabbit IgG) and heat treated in a thermocycler (Techne TC-PLUS) at 56 °C for 30 min followed by cooling to 20 °C for 15 min prior to analysis. Incubation of 5 μl bead array and 45 μl sample was performed at 20 °C on rotation (Grant) at room temperature over night. The beads were washed 3 × 100 μl PBST (0.05 % Tween-20) in a wash station (BioTek EL406) to remove unbound proteins, and the bound proteins cross-linked to the antibodies through addition of 0.4 % paraformaldehyde in PBS. After 10 min the beads were washed followed by incubation for 20 min with a streptavidin conjugated fluorophore (R-phycoerythrin, Invitrogen, SA10044 diluted 1:600 in PBST) serving as detection reagent. After a final wash, 100 μl PBST was added to the beads and the relative abundance of proteins measured in an LX200 or FlexMap3D instrument (Luminex Corp.). At least 34 beads per identity were required for statistical reliability and the median fluorescence intensity (MFI) per identity was used for data processing.

Serum samples were analyzed according to the protocol described previously [13]. In short, the samples were diluted 1:10 in PBS for labelling with a ten fold molar excess of biotin (total protein concentration approximated to 60 mg/ml) and was later diluted 1:50 in assay buffer.

### Sandwich assays

Concentrated BAL samples were diluted 1:80 for FN1 and 1:4 for CCL2 in PVXC and heat treated as for the direct labelling assay. The bead array with immobilized capture antibody was incubated with 25 μl of diluted sample over night. After incubation, the unbound proteins were washed off as described above, biotinylated detection antibody added at 1 μg/ml and incubation allowed for 1 h. The beads were then washed and incubated with the streptavidin conjugated fluorophore for 30 min before a final wash and readout in the FlexMap3D instrument. The FN1 detection antibody (MAB1918) was biotinylated through a bead-assisted procedure [15] while the CCL2 antibody was available in a biotinylated format as a component of a commercial sandwich ELISA kit (DY279, R&D).

### Statistical analysis

Statistical analysis and visualization were performed in R [16]. Data generated for BAL samples was analyzed as raw data while serum profiles were normalized using the Probabilistic Quotient Normalization (PQN) as described previously [17, 18]. Group comparisons were performed using Wilcoxon rank sum test and correlation coefficients based on Spearman correlation. Classification of samples based on protein levels was performed and visualised using receiver operating characteristic (ROC) curves (R package ROCR) using log transformed data for proteins either individually or combined through logistic regression.

## Results

**Fig. 1:**
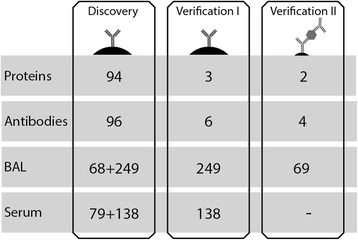
Overview of the experimental study design. The antibody suspension bead array technology was used to generate protein profiles in both BAL and serum. This study was performed as a directed screening using 94 selected target proteins. Towards these, 96 antibodies were used for profiling of both the discovery and verification set of BAL and serum samples. Based on these results, four proteins were selected for further analysis and additional antibodies targeting these proteins were included. As a second verification, the set of 68 samples were analyzed using sandwich assays for two of the proteins

In this study, we performed multiplex protein profiling in the search for proteins associated to sarcoidosis. Proteins previously linked to inflammation and disease pathogenesis were selected and profiled using the antibody suspension bead array technology. The initial setup consisted of 96 antibodies to 94 proteins that were applied for analysis of in total 317 BAL and 217 serum samples. Based on the results, three proteins were further analysed through additional antibodies and two of these also evaluated using sandwich immunoassays (Fig. 1).

### Differences in total protein concentration

Investigation of total protein content in the processed BAL samples revealed concentrations ranging from 0.2 to 9.8 mg/ml. The sarcoidosis patients displayed a higher median concentration than both the asthma patients and healthy controls (*p* < 1 × 10^−4^, Additional file 1: Figure S1). To avoid bias in protein detection deriving from these differences, the sample dilution buffer at labelling was supplemented with an excess of BSA to normalise the total protein content of the samples.

### Increased levels of FN1 and CCL2 in BAL fluid of sarcoidosis patients

**Fig. 2:**
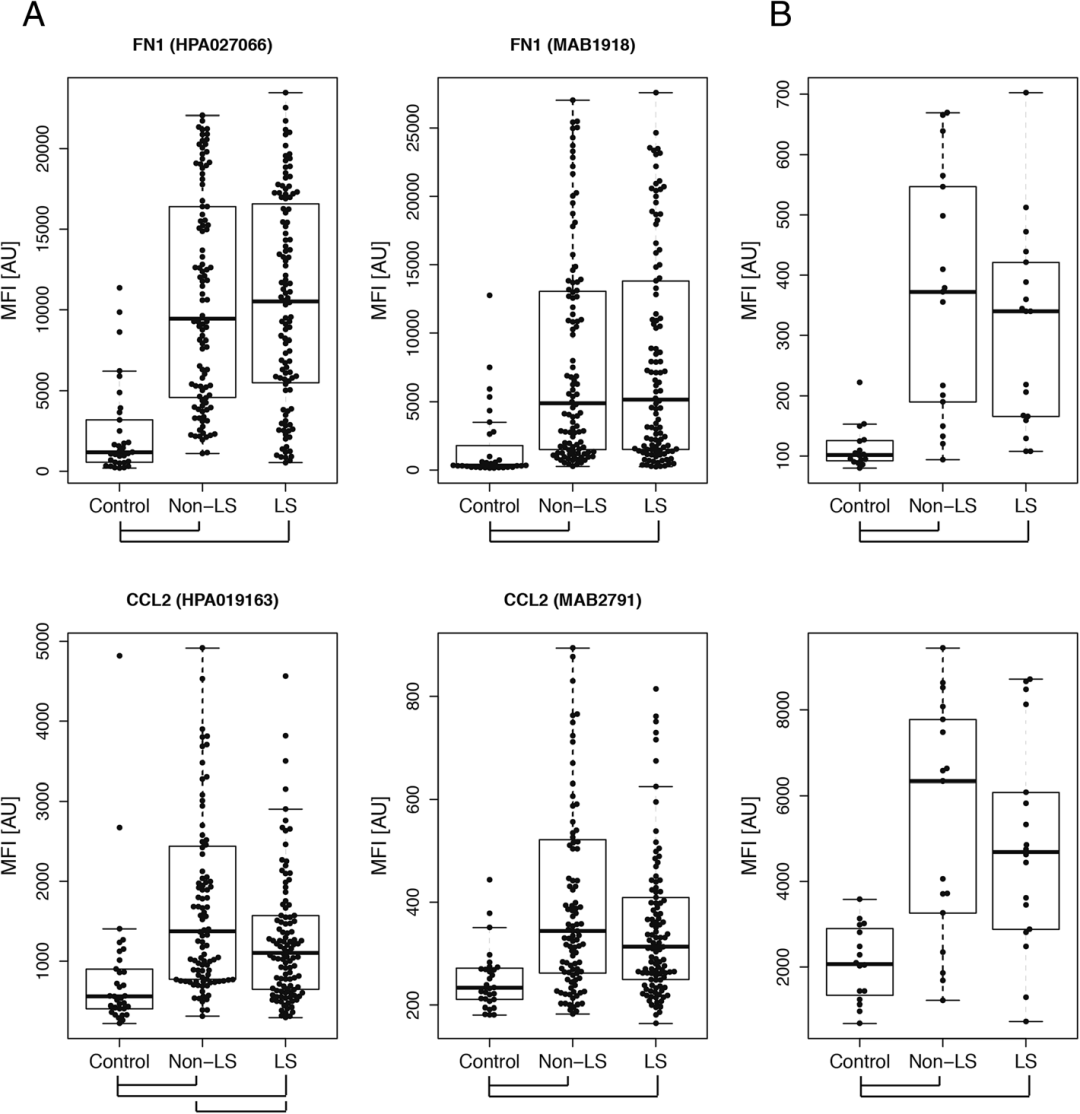
Levels of FN1 and CCL2 in BAL fluid. **a** The levels of FN1 and CCL2 were found as elevated in BAL from sarcoidosis patients compared to healthy controls using two independent antibodies. **b** These results were also verified using sandwich immunoassays. Outlier samples were excluded from the figure for visualization purposes and group differences with *p* < 0.05 are indicated

The 68 and 249 BAL samples were analysed in separate experiments using the 96plex bead array. The two datasets were compared and proteins with concordant differences between sarcoidosis patients and controls were identified. The two proteins with highest significance were fibronectin 1 (FN1, HPA027066) and C-C motif chemokine 2 (CCL2, HPA019163) with higher levels in the sarcoidosis group compared to the healthy controls (*p* < 0.001 in both sample sets). Additional proteins displaying differences in both data sets included cadherin 5 (CDH5, HPA030562), transferrin (TF, HPA005692), C-C motif chemokine 24 (CCL24, HPA035631), interleukin 15 (IL15, HPA037738), apolipoprotein A (LPA, HPA018130) and mitochondrial superoxide dismutase (SOD2, HPA001814). Higher levels of these proteins were also observed in the sarcoidosis group and p-values were below 0.01. For FN1 and CCL2, the differences were further evaluated in the first verification stage through profiling using additional antibodies, confirming the initial observation. The results for the 249 samples are visualised in Fig. 2a and Table 3 while the corresponding data for the 68 samples, where the asthma patients also displayed lower levels than the sarcoidosis patients (*p* < 0.01), is presented in Additional file 1: Figure S2. In addition to concordant group differences, the two antibodies for each protein also displayed positive correlations in terms of intensity levels (Rho > 0.64, Additional file 1: Figure S3). As a complement to the direct labelling approach, sandwich immunoassays were developed on the bead array platform. The set of 68 BAL fluid samples were analysed with this setup in a second verification stage, again confirming the initial results (Fig. 2b).

**Table 3:** Results from group comparisons of FN1 and CCL2 levels in BAL

			*P*-value			
Protein	Full name	Antibody	Sarc vs Control	LS vs Control	Non-LS vs Control	LS vs Non-LS
FN1	Fibronectin 1	HPA027066	4 × 10^−13^	3 × 10^−11^	3 × 10^−12^	0.8
		MAB1918	3 × 10^−10^	4 × 10^−9^	2 × 10^−9^	1
		Sandwich	3 × 10^−4^	2 × 10^−5^	7 × 10^−5^	0.5
CCL2	C-C motif	HPA019163	4 × 10^−7^	3 × 10^−5^	8 × 10^−8^	7 × 10^−3^
	chemokine 2	MAB2791	8 × 10^−6^	9 × 10^−5^	7 × 10^−6^	8 × 10^−2^
		Sandwich	1 × 10^−2^	2 × 10^−4^	3 × 10^−4^	0.7

*P*-values generated by two-group comparisons are presented together with the antibodies used for analysis

Although the BAL fluid levels of both FN1 and CCL2 were increased in sarcoidosis patients compared to both healthy and diseased controls, the levels of these two proteins were more similar between the two subtypes of disease, i.e., in LS and non-LS patients. For CCL2 however, we observed higher levels in non-LS patients with one of the antibodies in the larger sample material (HPA019163, *p* = 0.007). Comparison of protein levels between the subtypes of disease revealed no concordant findings for the remaining 94 proteins. In contrast to BAL fluid, the analysis of serum samples generated no concordant differences in protein levels reaching statistical significance.

**Fig. 3:**
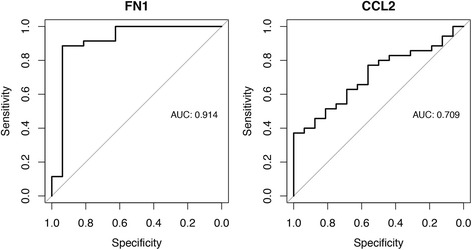
Classification of sarcoidosis patients using protein levels. ROC analysis was performed to determine how the levels of FN1 and CCL2 could classify sarcoidosis patients from controls. Logistic regression using the 249 samples as the training set was followed by testing of the 68 samples, as visualized in the figure

ROC analysis was applied to the data to evaluate how well the levels of FN1 and CCL2 could classify sarcoidosis patients from controls. Logistic regression was performed using the 249 samples as the training set followed by testing of the 68 samples. For the FN1 protein, area under the curve (AUC) values of 0.91 was obtained and for CCL2 a corresponding result of 0.71 (Fig. 3). Evaluation of the combined classification power of the two proteins revealed a discriminative potential similar to that of FN1 alone (AUC = 0.90). The lack of improvement compared to individual proteins could be explained by a positive correlation of FN1 and CCL2 levels in BAL (Additional file 1: Figure S4). Corresponding classification analysis of the two clinical subtypes of sarcoidosis revealed AUC values of 0.5 for FN1 and 0.6 for CCL2, illustrating the similarity in protein levels between the two subtypes of disease.

### Protein profiles associated to BAL fluid lymphocyte content

**Fig. 4:**
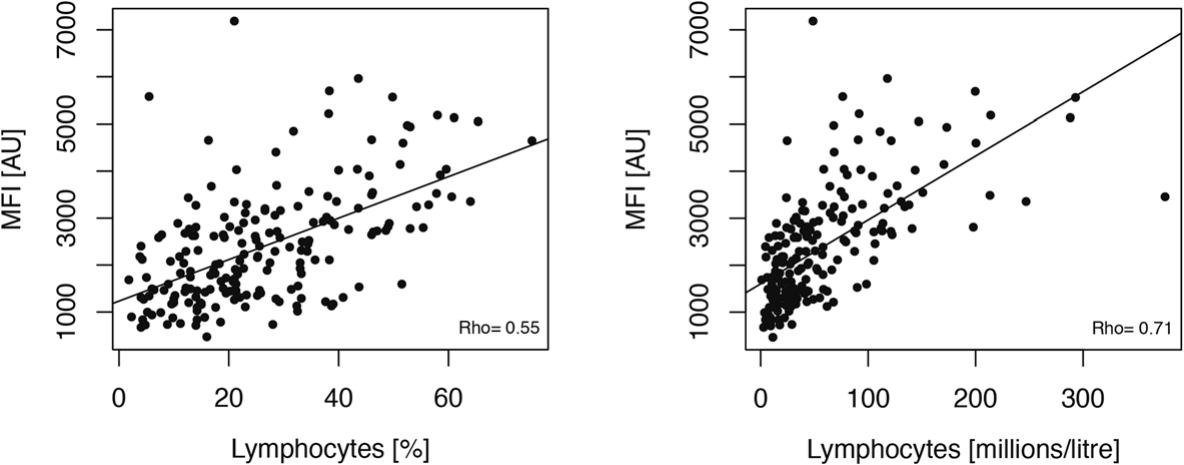
Correlation of CDH5 levels to BAL lymphocyte content. The level of CDH5 was found to correlate positively with the percentage of lymphocytes as well as the absolute lymphocyte concentration in BAL fluid

Besides the proteins found at different levels when comparing sarcoidosis patients with the control groups, levels of an additional protein was found interesting in relation to BAL cell differential counts. Cadherin 5 (CDH5, HPA030562) showed levels correlating positively to the percentage and cell count of lymphocytes present in BAL fluid (Rho = 0.55 and 0.71 respectively, see Fig. 4). In the initial verification stage of the study, these levels were also confirmed using an additional antibody (Additional file 1: Figure S3B). For other available parameters such as age, gender, chest radiograph stage, smoking, vital capacity (VC), forced vital capacity (FVC), forced expiratory volume in one second (FEV1), diffusion capacity of the lung for carbon monoxide (DLCO), and BAL content of other cell types including eosinophils, neutrophils, basophils, mast cells and the ratio of CD4/CD8 positive T-cells, no associations were observed.

### Correlation of protein levels in BAL and serum

As reported above, our data generated from serum analysis did not reveal any differences between either sarcoidosis patients and controls nor between the two sarcoidosis clinical subtypes. For individuals with paired BAL and serum samples, correlation analysis was performed to investigate the concordance in protein levels between the two sample materials. The two proteins with highest correlation coefficients in both sample sets were prostaglandin G/H synthase 2 (PTGS2, HPA001335, Rho 0.30 and 0.47) and napsin A (NAPSA, HPA026894, Rho 0.39 and 0.48) as presented in Additional file 1: Figure S5.

### Correlation to unprocessed BAL fluid

During the initial adaptation of the protocol for analysis of proteins in BAL fluid on suspension bead arrays, a small set of BAL samples (*n* = 8) were investigated in both unprocessed and concentrated forms. The results indicated that the concentration protocol was beneficial for detection of the majority of the 94 proteins investigated in this study. After selection of the three most interesting proteins, an extended evaluation was performed using a larger number of BAL samples from sarcoidosis patients (*n* = 69) and healthy controls (*n* = 5). FN1, CCL2 and CDH5 all revealed positive correlation of intensities between the unprocessed and concentrated sample materials (Additional file 1: Figure S6, Rho 0.75, 0.42 and 0.63 respectively).

## Discussion

Here we present a proteomic profiling approach using antibodies and bead arrays to investigate protein abundance in BAL and serum in order to find proteins associated to sarcoidosis. As a systemic inflammatory disease sarcoidosis can affect many body compartments, however the lungs are engaged in more than 90 % of patients. Hence BAL fluid is probably most suitable for identifying protein patterns of sarcoidosis. This study focused on proteins previously linked to sarcoidosis, lung function and inflammation resulting in a discovery phase screening 94 protein targets in 68 BAL samples from sarcoidosis patients, asthmatics and healthy controls. All targets were furthermore screened in 249 additional BAL samples from sarcoidosis patients and healthy controls, including additional antibodies towards three targets selected for further investigation.

Several proteins were identified with higher abundance in BAL of sarcoidosis patients, including cadherin 5 (CDH5), transferrin (TF), C-C motif chemokine 24 (CCL24), interleukin 15 (IL15) apolipoprotein A (LPA) and mitochondrial superoxide dismutase (SOD2). However, in the final analysis we focused on Fibronectin 1 (FN1) and Chemokine (C-C motif) ligand 2 (CCL2) that had the strongest associations to sarcoidosis in both sample sets. Fibronectin is a glycoprotein with a soluble form (plasma fibronectin) and an insoluble form (cellular fibronectin) found in plasma, extracellular matrix as well as on the cell surface [19]. It is involved in cell adhesion and migration processes, including wound healing, coagulation and host defence. Increased levels of fibronectin in BAL fluid have previously been associated with sarcoidosis and other interstitial lung diseases [20–23]. The concordance between our data and these studies, where FN1 was measured by ELISA, confirm the suitability of our assay for protein analysis in BAL. Furthermore, our results showing no FN1 differences in serum of sarcoidosis patients compared to healthy controls mirror previous results in plasma [21]. Currently, elevated BAL levels of fibronectin is suggested as a marker of lung inflammation rather than reflecting sarcoidosis specific processes [20, 24, 25]. Nevertheless, the small set of asthma patients included in this study displayed lower levels of FN1 compared to the sarcoidosis groups and were in line with the healthy controls.

Chemokine ligand 2 (CCL2), also known as monocyte chemoattractant protein 1 (MCP-1), is a cytokine recruiting monocytes, memory T cells and NK cells to sites of active inflammation and has been implicated in the pathogenesis of other inflammatory diseases such as atherosclerosis, RA and multiple sclerosis [26]. We found increased levels of CCL2 in BAL fluid of sarcoidosis patients compared to controls, which is in line with previous studies [27, 28] although there are contradictory reports as well [29]. Palchevskiy et al. found elevated BAL levels of CCL2 and CCL5 throughout all stages of sarcoidosis [27], but while Christophi et al. found increased mRNA levels of CCL2 in tissue samples from sarcoid granulomas, the CCL2 levels could not differentiate sarcoid granulomas from other granulomatous inflammation [30]. In our study, we could not find altered levels of CCL2 in serum, which is in line with recent results [7].

The availability of extensive clinical information is vital for this kind of study as it allows for correlation of protein patterns beyond healthy/disease. In this study, cadherin 5 (CDH5) was found to correlate positively with BAL lymphocyte counts of both patient cohorts. However, no association was found to the other available parameters including smoking. As sarcoidosis is considered to be a Th1-driven disease characterized by an accumulation of lymphocytes in BAL fluid, CDH5 levels as a measure of lymphocyte infiltration could potentially be used to monitor disease progression. Furthermore, ROC analysis separating patients from healthy controls with AUC values of 0.91 and 0.71 for FN1 and CCL2 respectively, indicated applicability for clinicians in ambiguous patient cases. To that end, future studies evaluating these proteins in relation to other interstitial lung diseases are needed.

Interestingly, none of the proteins analysed revealed concordant differences in the two sample sets between the subtypes of sarcoidosis, namely Löfgren’s syndrome (LS) and non-Löfgren’s syndrome (Non-LS). These groups of patients differ not only in their clinical symptoms and disease progression but have also been found to display differential genetic profiles [31]. Even so, they also share common characteristics, including granuloma formation, which could associate with similar protein expression patterns in the local environment of the lung. The patient population included in this study consisted mainly of patients with early stages of disease as indicated by the high representation of subjects classified with chest radiographic stage I and II. However, we do not see this as a major limitation of the study as the intended aims included whether protein profiles could provide potential markers to support diagnosis and monitoring of disease progression or treatment response – all of which focus on the earlier stages of disease. Therefore, a clinically useful marker would either predict disease course already at disease onset or help set the diagnose, rather than reflect protein expression at a later stage of the disease. In addition, patients at later stages are usually on treatment, potentially influencing several aspects of the pulmonary inflammation most likely including protein expression. Nonetheless, further insight into overall disease pathogenesis would have benefited from more patients in stages III and IV.

In this study, we observed higher levels of total protein concentration in BAL from sarcoidosis patients compared to healthy controls as well as asthma patients. Such difference could potentially influence protein analysis, thus it is important to elucidate whether this has a technical/processing or biological origin. Since the BAL samples were concentrated prior to analysis, we chose to complement our initial analysis and also include 74 of the samples in an unprocessed state. Although all proteins could not be detected in crude sample material, which in turn supports the use of concentrated material, the high correlations of FN1 and CDH5 indicates that the differences in total protein concentration are not due to variability in the concentration procedure but rather of biological origin. Furthermore, sarcoidosis patients have previously been reported with elevated BAL levels of albumin, suggested to be related to the chronic inflammation and not the lavage procedure itself. This explains the differences in total protein concentration as of biological origin and importantly shows that normalizing based on measured total protein or albumin concentrations might mask disease related differences in the analysis phase [28, 29].

The most abundant proteins in BAL fluid are plasma proteins (albumin, immunoglobulins, etc.) thought to be derived from diffusion across the blood-air barrier [32]. As implied earlier, interstitial lung diseases such as sarciodosis, lead to increased BAL fluid levels of plasma proteins, however there is no consensus on the underlying mechanisms (altered barrier permeability, active transport or local production) [32–34]. In our recent evaluation of autoimmune IgG specificities in sarcoidosis, results were highly concordant between BAL and serum with correlation coefficients above 0.81 [35]. A bi-directional transcytosis mediated by the Fc-gamma receptor was the suggested mechanism for transport across the epithelium. Thus IgG produced locally in the lung is spread systemically and circulating IgG will be present in the lung environment. Looking at the total set of 94 proteins investigated in this study, individual protein levels in BAL compared to serum had weak to moderate correlations (up to Rho 0.48). This implies a minor role for a general increased barrier permeability causing the increased plasma protein content of BAL from sarcoidosis patients. The fact that serum levels of the 94 investigated proteins could not differentiate sarcoidosis patients from controls further indicate that differences seen in BAL are the results of local processes in the lung or active transport rather than reflecting a systemic state.

## Conclusion

Previous proteomics studies in the field of sarcoidosis have typically investigated BAL or serum from a limited number of patients, applying differential proteomic analysis through 2D-electrophoresis, using MS to identify proteins in the spots of interest [36–39]. There have been recent efforts to study the intracellular proteome of alveolar macrophages [8]. Previous reports of multiplex screening for proteins of interest in sarcoidosis have included up to 30 targets [7] but here we present a analysis of 94 different targets with an assay potential of 384 markers screened in 384 samples per assay. The flexibility of our assay and the resources of the Human Protein Atlas provide an exceptional opportunity to perform targeted screening of hundreds of selected protein targets in hundreds of biological fluid samples (BAL, serum, plasma, etc.). The latter is very important as investigating many variables in a limited set of samples pose statistical challenges in the data analysis. The ability to simultaneously screen a wide range of samples, preferably from multiple cohorts and diseases, increases the probability of identifying proteins of relevance for further investigations of potential utility in clinical practise.

## Abbreviations

BAL, bronchoalveolar lavage; BSA, bovine serum albumin; CCL2, C-C motif chemokine 2; CCL24, C-C motif chemokine 24; CDH5, cadherin 5; FN1, fibronectin; HPA, human protein atlas; IL15, interleukin 15; LPA, apolipoprotein A; LS, Löfgren syndrome; MFI, median fluorescence intensity; SOD2, mitochondrial superoxide dismutase; TF, transferrin.

## Additional file

Additional file 1:
**Figure S1.** Total protein concentration. **Figure S2.** Levels on FN1 and CCL2 in BAL. **Figure S3.** Correlation of paired antibodies. **Figure S4.** FN1 and CCL2 correlation. **Figure S5.** Correlations of protein levels in BAL fluid and serum. **Figure S6.** Correlations of protein levels in unprocessed and concentrated BAL. (DOCX 685 kb)
