# Supplementary material for: Elevated levels of FN1 and CCL2 in bronchoalveolar lavage fluid from sarcoidosis patients
Source: Respir Res. 2016 Jun 4;17:69. doi: 10.1186/s12931-016-0381-0 (PMC4893268; doi:10.1186/s12931-016-0381-0)

# Supplementary information

## Figure legends

### Supplementary figure 1. Total protein concentration

Total protein concentration of BAL samples included in the study revealed higher protein content in samples from sarcoidosis patients compared to both healthy controls and asthma patients (p < 0.0001).

### Supplementary figure 2. Levels on FN1 and CCL2 in BAL

Levels of the two proteins in the set of 68 BAL samples with indications of group differences with p-values < 0.05.

### Supplementary figure 3. Correlation of paired antibodies

(A) Correlation of the two antibodies included for the three selected proteins indicated concordant results. (B) Schematic representation of antigens used for antibody production compared to the corresponding native proteins.

### Supplementary figure 4. FN1 and CCL2 correlation

Correlation of FN1 and CCL2 levels in the 249 BAL samples.

### Supplementary figure 5. Correlations of protein levels in BAL fluid and serum

The two proteins with highest correlation between the two body fluids were NAPSA and PTGS2. Results are shown for paired samples in A) the initial sample material and B) the extended sample collection.

### Supplementary figure 6. Correlations of protein levels in unprocessed and concentrated BAL

The levels of the three selected proteins showed positive correlation in unprocessed and concentrated BAL.

## Figures

### Supplementary figure 1. Total protein concentration


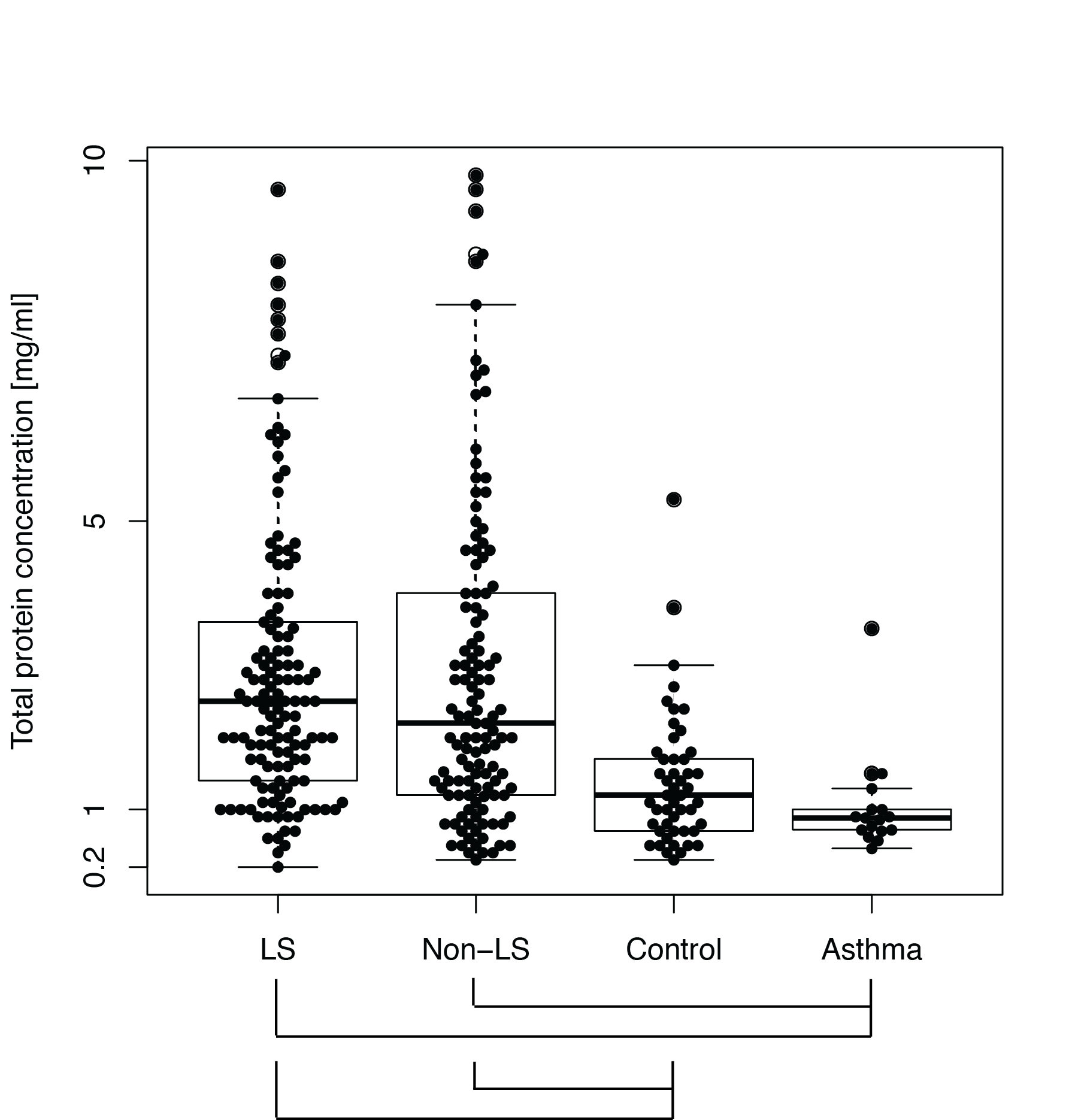


### Supplementary figure 2. Levels of FN1 and CCL2 in BAL


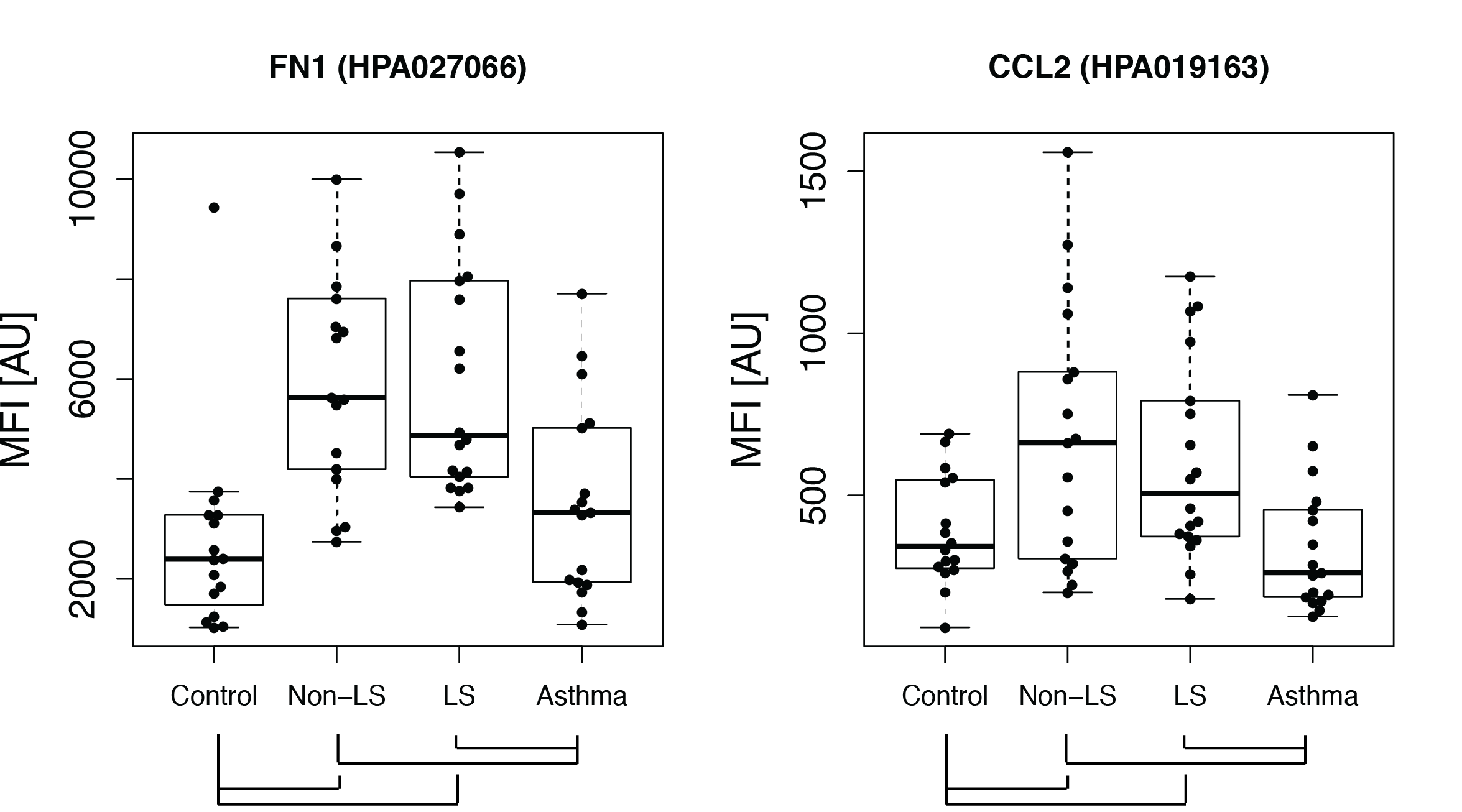


### Supplementary figure 3. Correlation of paired antibodies


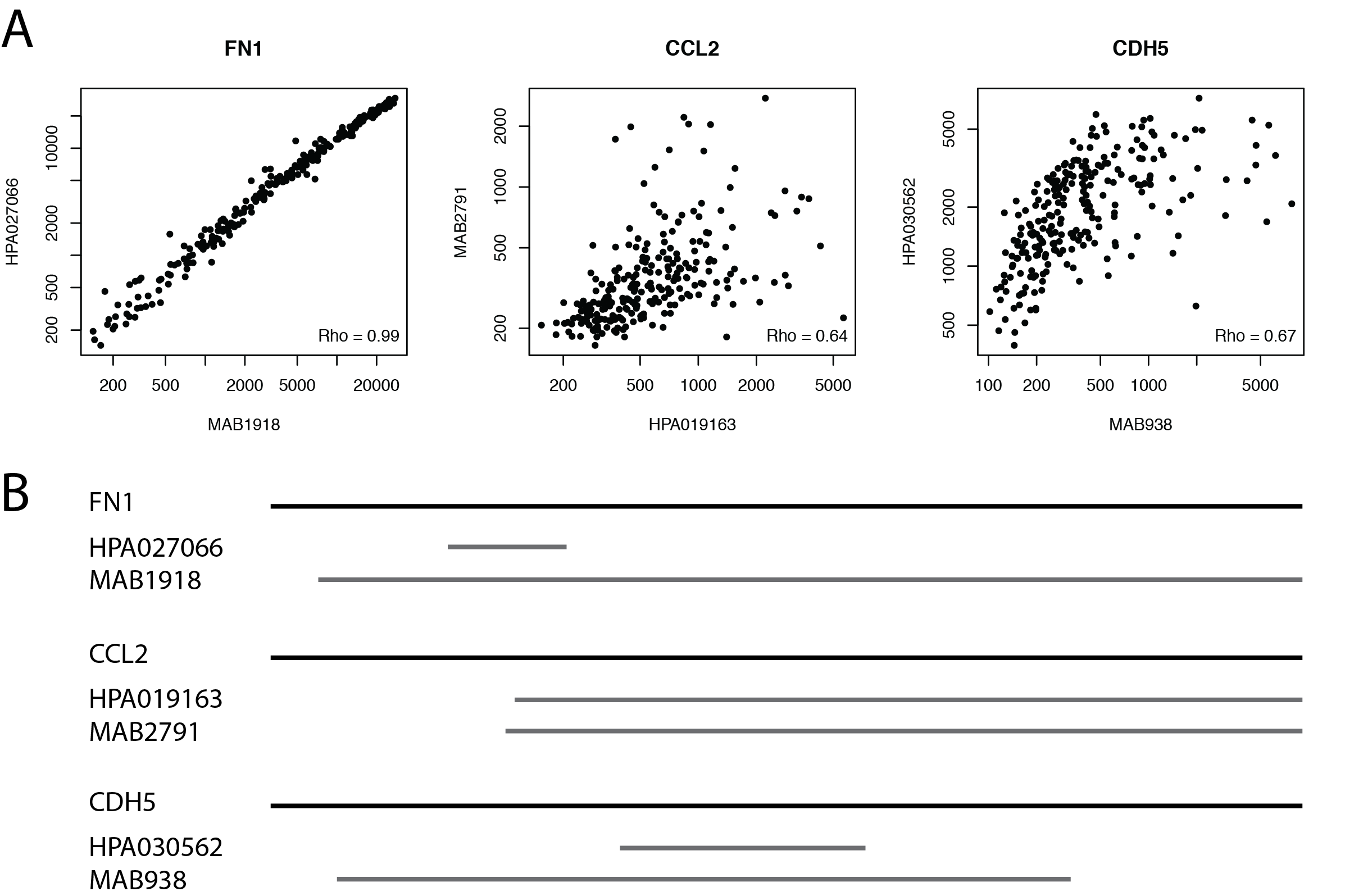


### Supplementary figure 4. FN1 and CCL2 correlation


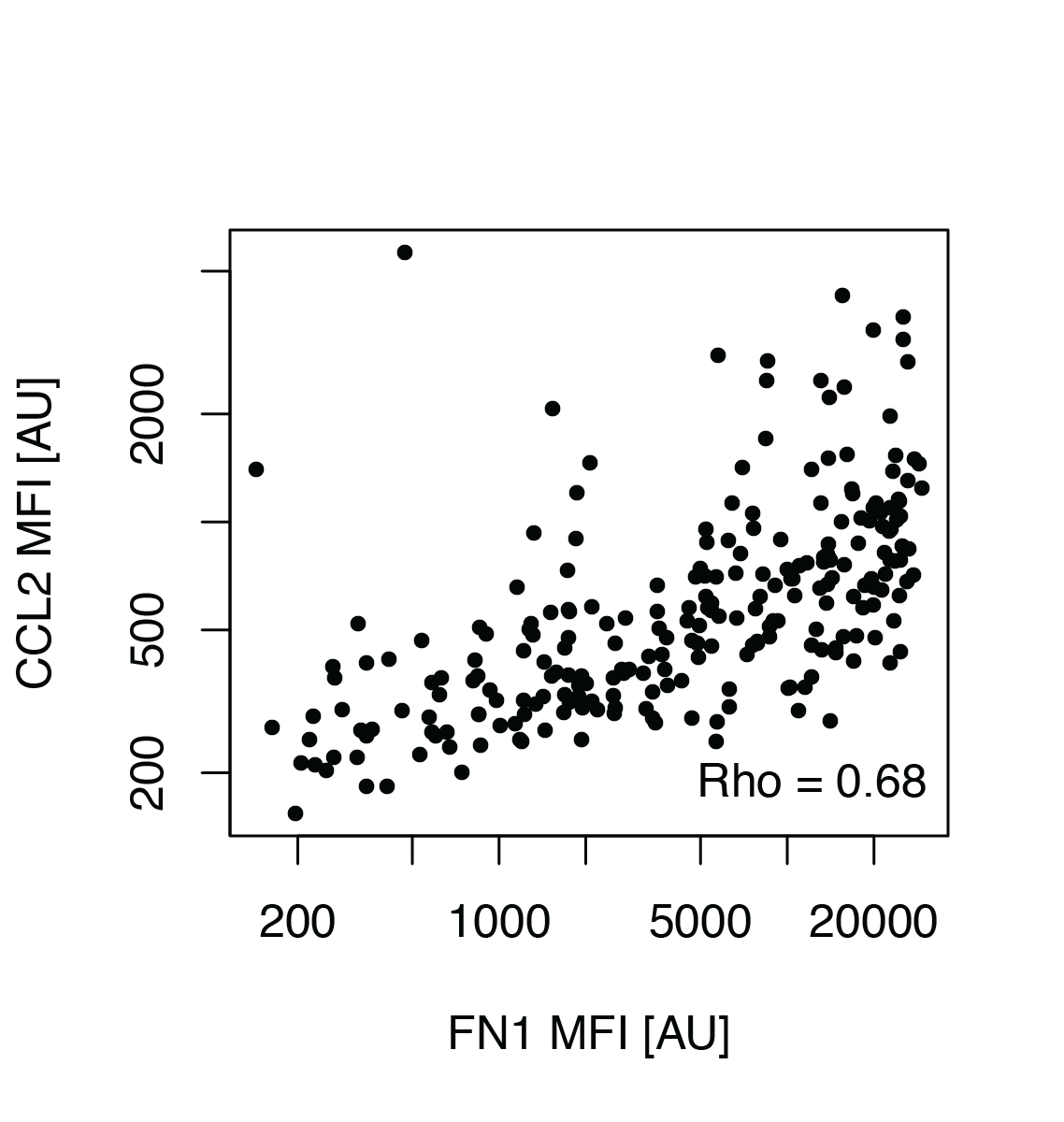


### Supplementary figure 5. Correlations of protein levels in BAL fluid and serum


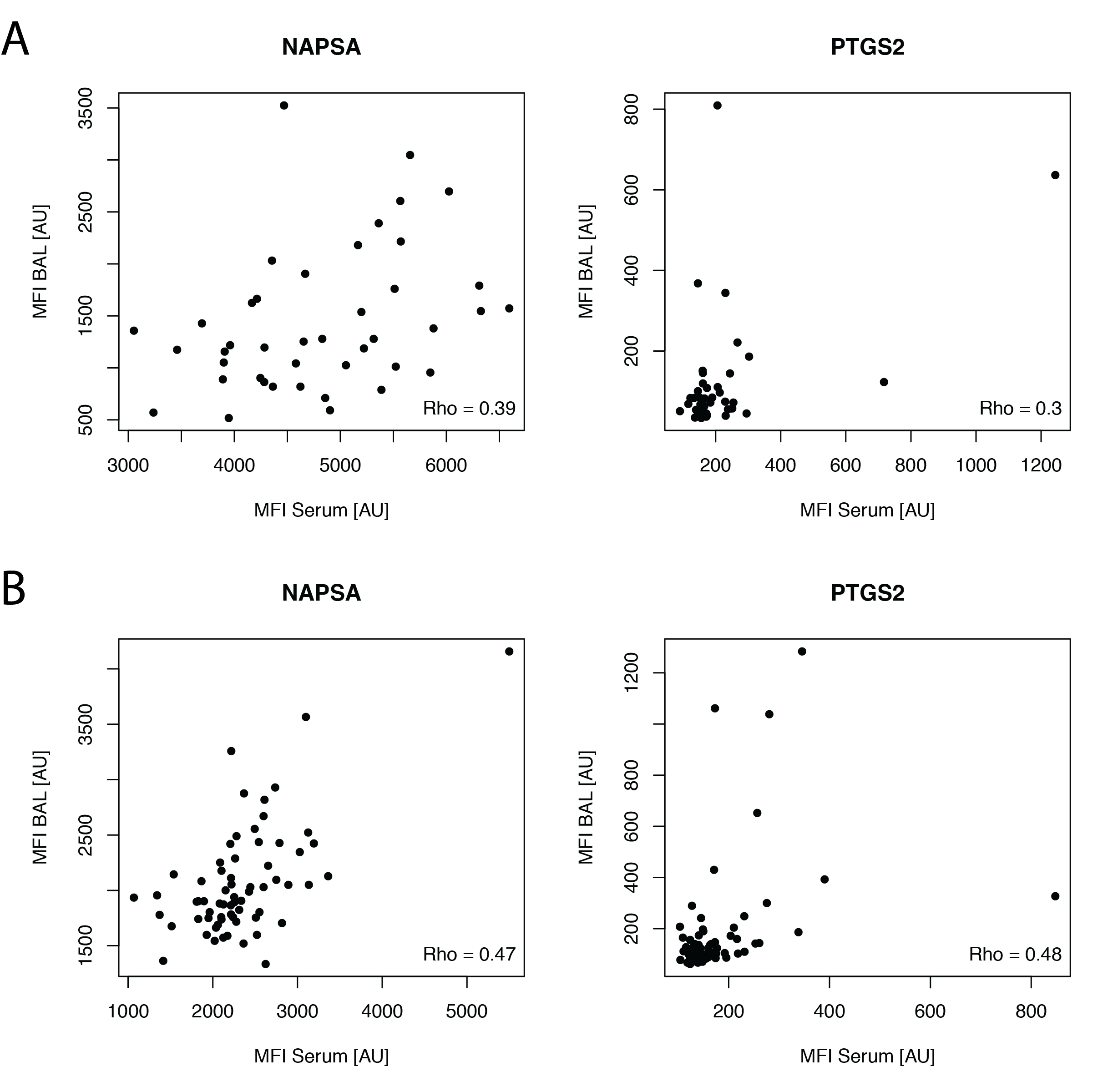


### Supplementary figure 6. Correlations of protein levels in unprocessed and concentrated BAL


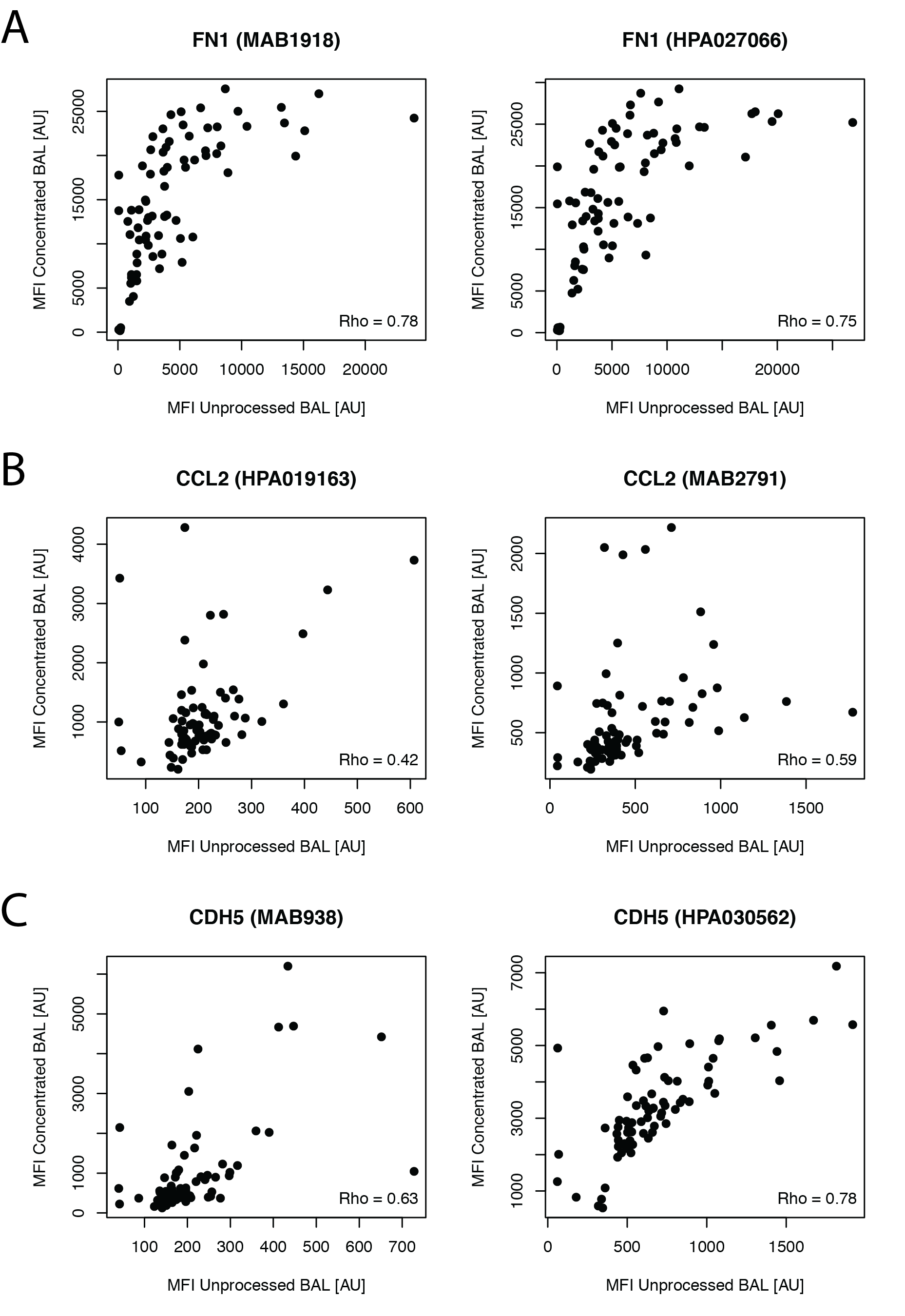

Supplement: Additional file 1: — Figure S1. Total protein concentration. Figure S2. Levels on FN1 and CCL2 in BAL. Figure S3. Correlation of paired antibodies. Figure S4. FN1 and CCL2 correlation. Figure S5. Correlations of protein levels in BAL fluid and serum. Figure S6. Correlations of protein levels in unprocessed and concentrated BAL. (DOCX 685 kb) [file 12931_2016_381_MOESM1_ESM.docx]
